# A Novel Hybrid Error Criterion-Based Active Control Method for on-Line Milling Vibration Suppression with Piezoelectric Actuators and Sensors

**DOI:** 10.3390/s16010068

**Published:** 2016-01-06

**Authors:** Xingwu Zhang, Chenxi Wang, Robert X. Gao, Ruqiang Yan, Xuefeng Chen, Shibin Wang

**Affiliations:** 1 State Key Laboratory for Manufacturing System Engineering, School of Mechanical Engineering, Xi’an Jiaotong University, Xi’an 710049, China; xwzhang@mail.xjtu.edu.cn (X.Z.); wangchenxi@stu.xjtu.edu.cn (C.W.); wangshibin2008@mail.xjtu.edu.cn (S.W.); 2 Department of Mechanical and Aerospace Engineering, Case Western Reserve University, Cleveland, OH 44106, USA; robert.gao@case.edu (R.X.G.); ruqiang@seu.edu.cn (R.Y.); 3 School of Instrument Science and Engineering, Southeast University, Nanjing 210096, China

**Keywords:** active control, hybrid error criterion, milling process, vibration suppression

## Abstract

Milling vibration is one of the most serious factors affecting machining quality and precision. In this paper a novel hybrid error criterion-based frequency-domain LMS active control method is constructed and used for vibration suppression of milling processes by piezoelectric actuators and sensors, in which only one Fast Fourier Transform (FFT) is used and no Inverse Fast Fourier Transform (IFFT) is involved. The correction formulas are derived by a steepest descent procedure and the control parameters are analyzed and optimized. Then, a novel hybrid error criterion is constructed to improve the adaptability, reliability and anti-interference ability of the constructed control algorithm. Finally, based on piezoelectric actuators and acceleration sensors, a simulation of a spindle and a milling process experiment are presented to verify the proposed method. Besides, a protection program is added in the control flow to enhance the reliability of the control method in applications. The simulation and experiment results indicate that the proposed method is an effective and reliable way for on-line vibration suppression, and the machining quality can be obviously improved.

## 1. Introduction

With the development of manufacturing application requirements, machining quality and precision are more critically required nowadays. However, processing vibration seriously affects the stability of cutting processes, and hence machining quality and precision. Kayhan *et al.* compared the machining quality between two components, in which the quality of one component is seriously reduced by chatter vibration [1]. Novakov *et al.* indicated by experiment that the chatter vibration will reduce the lifetime of cutting tools about 50%–80% [2]. Therefore, it’s significant to control the machining vibration to improve the machining quality and reliability of machine tools.

**Figure 1:**
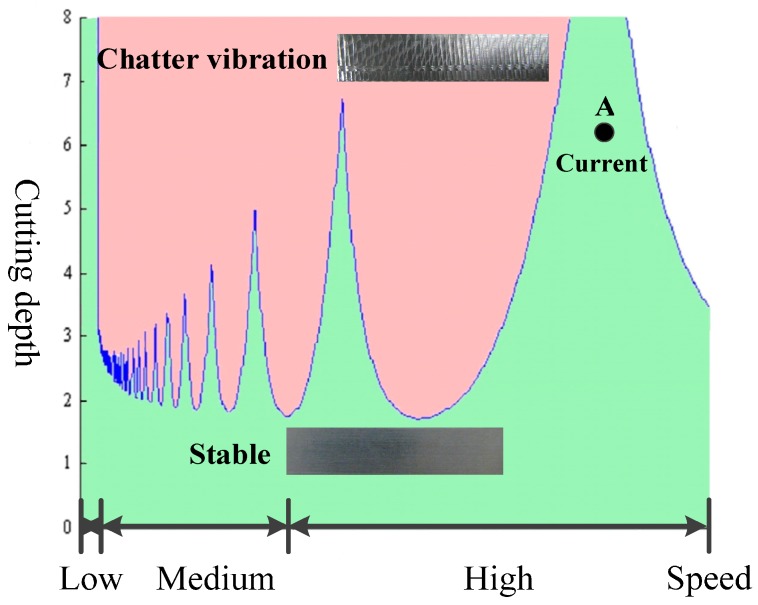
Cutting lobe of the machining process.

There are mainly three methods for decreasing the influence of cutting vibration. The first one is to adjust the processing parameters in a stable region. As shown in Figure 1, the machining condition can be adjusted by changing the cutting depth and spindle speed.

If the processing state is located in the green region, *i.e.*, point A, the machining process will be stable and the machining quality will be satisfactory. However, if the processing state is located in the red region, the machining process will fail due to the chatter vibration. Unfortunately, nobody can guarantee the whole machining process will always located in the stable region, because it relies on experience and the processing state varies during the whole machining process.

The second method is to optimize the structures and passive vibration control. For example, in order to guarantee processing quality, some thin wall components in aerospace engineering are manufactured by real-time cutting compensation. That is, the influences of the deformation will be simulated precisely first and then an opposite cutting compensation set in the machining process. However, this needs a very precise and expensive simulation with low-adaptability. The third method is real-time active vibration control, which can accomplish on-line control of the cutting vibrations to guarantee the machining process to be stable. It is an adaptive and efficient way to deal with this problem, and thus has attracted many researchers’ and engineers’ attention [3,4]. Due to their excellent vibration reduction performance, active vibration control methods have been applied in many industrial areas, such as machine tools, airplanes, ships, cars and hard disks, *etc.* Taking machine tools as an example, Kakinuma implemented chatter vibration suppression by developing a hybrid control method and verified this method by experiments [5]. Hesselbach achieved noise and vibration reduction for machining of composite boards by an active clamping system based on piezo-stack actuators, although a special work fixture is needed. [6]. Monnin proposed two different optimal control strategies and applied chatter vibration control for milling processes [7,8]. Long developed an active vibration control system based on feedback controller synthesis with a robust mixed sensitivity method for peripheral milling processes [9]. Xu achieved field balancing and harmonic vibration suppression for high-speed rotors based on active magnetic bearings and two control methods, synchronous current reduction approach and repetitive control algorithm [10].

Apart from machine tools, active control methods have also been applied in other mechanical engineering areas and obtained satisfactory effects. For example, in the area of vehicles, Kwak proposed a hardware-in-the-loop system to estimate the efficiency of active vibration control of lateral vibrations of railway vehicles by a magneto-rheological fluid damper [11]. Nguyen implemented a semi-active vehicle seat-suspension system based on a novel neuro-fuzzy controller and achieved a satisfactory performance verified by experiments [12,13]. Sun investigated the problem of vibration suppression in vehicular active suspension systems by simulation, in which the adaptive robust control strategy is used to realize the disturbance suppression [14]. Li investigated the adaptive sliding mode control problem for nonlinear active suspension via the Takagi-Sugeno fuzzy approach [15].

Military equipment and other basic applications have also been explored. Daley introduced a new hybrid active/passive mounting system in vibration reduction of large marine machinery rafts [16]. Structural vibration and structure-borne noise in water of a submerged finite cylindrical shell is investigated by an active vibration method based on macro fiber composites and an optimal control algorithm [17]. Zhang constructed a dynamic frequency characteristics active control method for vibration optimization of beam-plate systems and scaled underwater vehicle models [18,19]. Chamroon investigated active vibration control in multimode rotor-dynamic systems based on dynamic strain feedback and an optimal model-based controller synthesis [20]. Li presented an active control simulation of the acoustic and vibration response of a vibro-acoustic cavity of an airplane based on a PID controller and an Eulerian model [21]. Lin proposed a self-organizing fuzzy controller for active suspension systems, in which the optimal parameters could be obtained by the developed hybrid self-organizing fuzzy and radial basis function neural network controller [22]. Ferrari applied a positive position feedback algorithm in active vibration control of single-input single-output and multi-input multi-output systems and successfully mitigated the vibration of the first four natural modes of a sandwich plate [23]. Sun proposed a novel 3-D quasi-zero-stiffness system and applied it in active vibration control with satisfactory results [24]. Wu studied the accelerometer configuration measurement model for active control of vibration isolation platforms [25]. Her implemented vibration analysis of composite laminate plates bonded with piezoelectric patches for active control systems [26].

Although many control algorithms have been developed and many practical achievements have been made for vibration control, the efficiency, adaptability and anti-interference ability need to be addressed further. Since time-domain control algorithms are mainly used in these systems, the real-time performance is strict both for the control algorithm and hardware devices. Besides, one single time-domain error criterion is used to determine the control process. Therefore, it is easy to make the control process drop into local optimum results with less adaptability and anti-interference ability. In this paper, a novel hybrid error criterion frequency-domain LMS active control method is proposed to enhance the efficiency, adaptability and anti-interference ability of this kind of method in on-line vibration suppression, and verified on a milling process. First, the control algorithm is constructed in the frequency domain, so it is not sensitive to the real time performance. Then the correction formulations of the weights are derived by the steepest decent procedure. Third, a hybrid error criterion is proposed to improve the adaptability and anti-interference ability of the control algorithm and system. Finally, the control method is verified and investigated by simulation and experiments, and a protection program is added to enhance the reliability of this method in applications.

## 2. Control Algorithm Design

LMS [27] is a classical and typical adaptive signal processing method, whose control scheme is simple and efficient, so it’s suitable for real-time active vibration control. Compared with the time-domain LMS control method, the frequency-domain LMS is not sensitive to the transient response and suitable for active vibration control with a periodic response, so the frequency-domain method is chosen as the core control scheme in this paper. Different from the traditional LMS method, the frequency-domain LMS constructed in this paper has three advantages. First, only one Fast Fourier Transform (FFT) is used in each control cycle and no Inverse Fast Fourier Transform (IFFT) is involved. Second, the control parameters are optimized to improve the efficiency. Third, a new hybrid error criterion is constructed to enhance the adaptability, robustness of the frequency-domain LMS method.

### 2.1. Control Scheme

**Figure 2:**
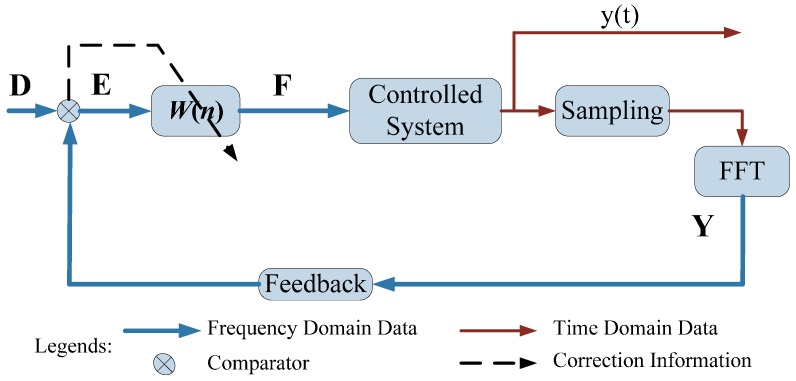
Frequency-domain LMS control scheme.

The frequency-domain LMS control scheme for active vibration control is shown in Figure 2, where, **D** is the frequency-domain destination signal. **E** is the error signal. **F** represents the optimized actuating parameters. y(t) is the time-domain vibration response and **Y** is the frequency-domain vibration response. “Sampling” represents the vibration data collection process. The control process runs according to the following steps. First, a vibration response y(t) is collected by “Sampling” at a determined sampling rate. Then the time-domain response y(t) is transformed to a frequency-domain signal **Y** by FFT. The difference between **Y** and **D** is then set as input for LMS controller to update the weights *W*(*n*). Next, the optimized actuating parameters **F** are sent to the control system as the secondary vibration source to suppression the original vibration. This control cycle is repeated until the difference between **D** and **Y** satisfies a preset precision. It can be seen from the control flow that the FFT is only used once in each cycle and no IFFT is introduced, so the control flow is simplified and the control time is reduced.

In order to make the control algorithm converge to the target solution, the frequency error and the iterative functions should be constructed. The global frequency error **J** in this control scheme to be minimized by adjusting weights is defined as:

(1) $$J=\frac{1}{2}\sum_{i=1}^{n}{\left(d_{i}-y_{i}\right)}^{2}=\frac{1}{2}{\left\|D-Y\right\|}^{2}$$

where, *d_i_* is the *i*th element of the destination signal **D**. *y_i_* is the *i*th element of the frequency-domain vibration response **Y**.

The global frequency error **J** in Equation (1) represents the difference between the target signal and the vibration response in the whole concerned frequency range. The control algorithm can be effective only in the condition of the frequency error **J** is to be diminished smoothly in the control process. Therefore, the iterative equation of the weights is constructed to guarantee the convergence. The steepest descent method is used to obtain the iterative equation of the weights *w*(*n*) of controller:

(2) w(n+1)=w(n)−ηΔw(n)

where, *η* is the learning rate and represents the step size. *w*(*n* + 1) is the weights in step *n* + 1 and *w*(*n*) is the weights in step *n*. Δ*w*(*n*) is the gradient vector.

By taking a derivation to *w*(n) of **J**, the variation of the weights Δ*w*(*n*) in each step can be obtained to guarantee the convergence:

(3) $$\Delta w(n)=\frac{\partial J}{\partial w(n)}=\frac{\partial (\frac{1}{2}{\left\|D-Y\right\|}^{2})}{\partial w(n)}=\frac{\partial (\frac{1}{2}e^{2}(n))}{\partial w(n)}=-e^{2}(n)$$

where, $e(n)=\sum_{i=1}^{m}\left(d_{i}(n)-y_{i}(n)\right)$ is the global frequency error in *n*th iterative step.

Substituting Equation (3) into Equation (2), the iterative equation of the weights *w*(*n*) can be obtained as follows:

(4) $$w(n+1)=w(n)+\eta e^{2}(n)$$

### 2.2. Error Criterion

In order to improve the adaptability, anti-interference ability and reliability of the control algorithm, a new error criterion is constructed. The global frequency error **J** in Equation (1) is used to update the weights of the control network, while the frequency node error **J**_n_ in Equation (5) is combined together with **J** as the error criterion in Equation (6):

(5) $$J_{n}=\frac{1}{m}\sum_{j=1}^{m}{\left(d_{j}-y_{j}\right)}^{2}$$

where, *m* denotes the number of characteristic frequencies. *d_j_* is the amplitude of the *j*th characteristic frequency in the destination signal **D**, and *y_j_* is the amplitude of the *j*th characteristic frequency in the frequency-domain vibration response of **Y**:

(6) $$J\;\leq \;pre\_g\text{ \& }J_{n}\;\leq \;pre\_n$$

where, *pre_g* is the convergence precision of the global frequency error, and *pre_n* is the convergence precision of the frequency node error.

**Figure 3:**
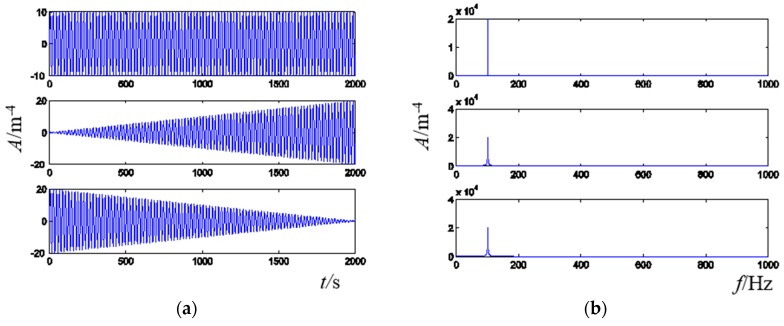
Illustration of the relationship between time-domain signals and frequency-domain signals. (**a**) Time-domain signal; (**b**) Frequency-domain signal.

There are two advantages of the error criterion constructed for the presented active vibration control method in the frequency domain. First, the control efficiency can be improved and the control process will be more smooth. The global frequency error is used for weight updating in each step of the presented method and the objective signal and the real-time vibration signal are all in the frequency domain, so only one FFT is used for vibration response in each iteration, and no IFFT is included, thus control time is saved. Furthermore, as shown in Figure 3, the time-domain signal sometimes is not in a one-to-one correspondence with the frequency-domain signal, so the global frequency error can precisely reflect the difference between the frequency-domain real-time response and objective. Otherwise, the control process may oscillate if the time-domain error is used in this situation.

**Figure 4:**
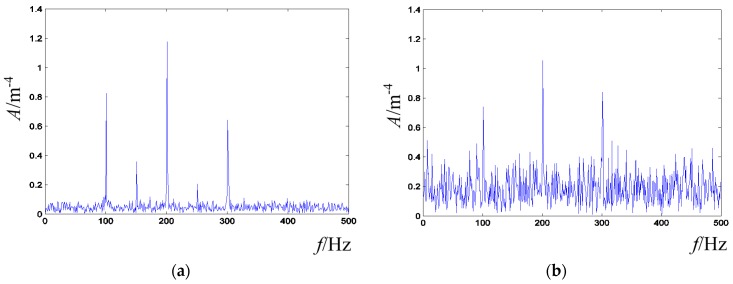
Illustration of the unacceptable case that may occur if the frequency node error is alone taken as the error criterion. (**a**) Destination signal; (**b**) Vibration signal.

Second, the proposed error criterion can enhance the adaptability and anti-interference ability of the presented active control method. In many cases, we always focus on the characteristic frequency node amplitude, if the global frequency error **J** is alone taken as the error criterion, which cannot efficiently effect the changes on these characteristic frequency nodes. However, if the frequency node error **J_n_** alone is taken as the error criterion, some unacceptable cases, such as that illustrated in Figure 4, may be obtained. Therefore, the global frequency error **J** and frequency node error **J_n_** are combined together as the error criterion, and the adaptability of the constructed method can be enhanced by appropriately adjusting the *pre_g* and *pre_n* in Equation (8). Besides, since the frequency node may vary slightly in practical cases, the anti-interference ability can be enhanced by setting a variation range for frequency nodes in the error criterion.

## 3. Simulation and Experimental Verification

**Figure 5:**
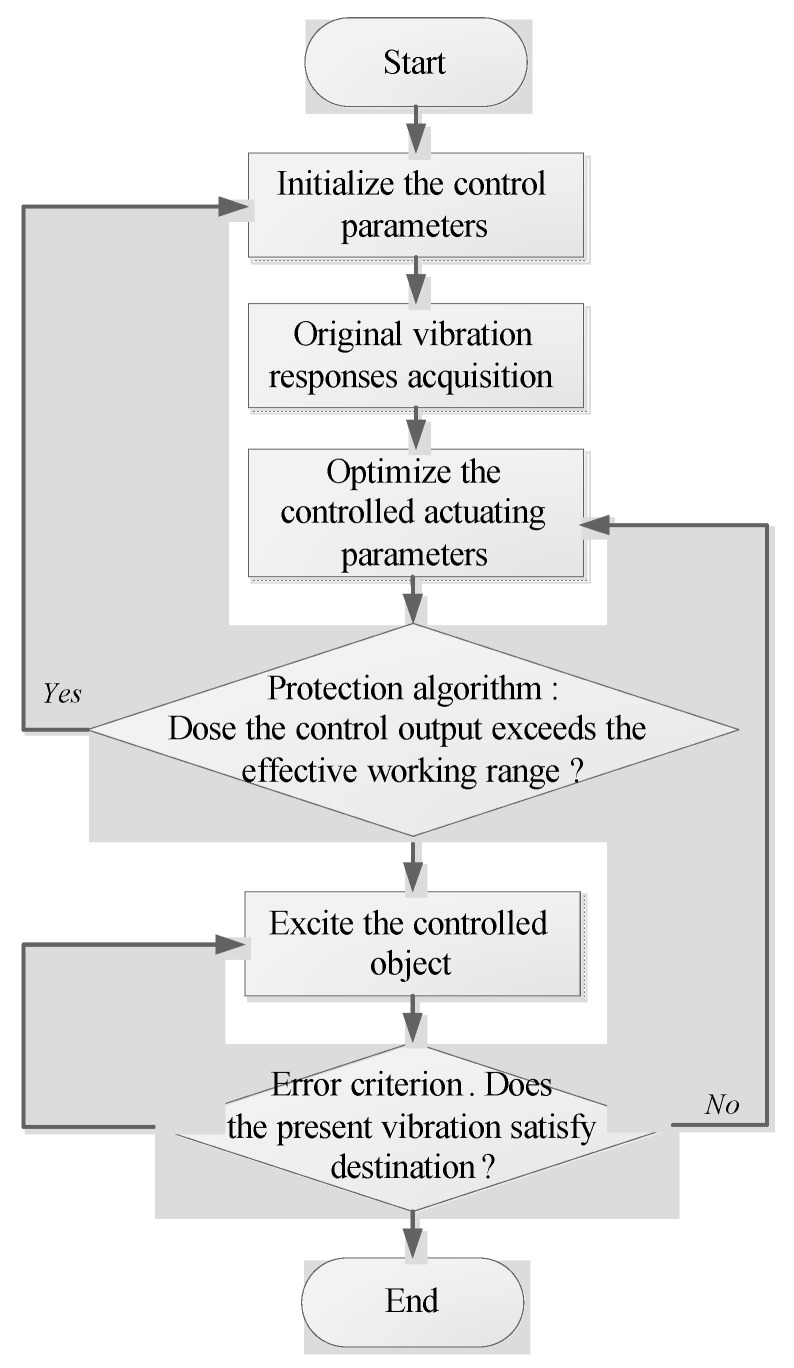
Flow chart of the control process.

In order to verify the effectiveness of the proposed control method, simulations and experiments are presented in this section. The flow chart of the control method is shown in Figure 5. A special point that should be mentioned is the “protection algorithm”, which is added in this algorithm to make it more reliable and safe. The control process may diverge due to some practical reasons and the control parameters may exceed the working range, thus the devices may be destroyed by these inappropriate parameters. However, the “protection algorithm” can avoid these losses in such cases. It will analyze the error in every iterative step, and set the initialized control again with new parameters if the control algorithm works in a wrong convergence tendency or inappropriate control parameters.

### 3.1. Spindle Based Simulation

**Figure 6:**
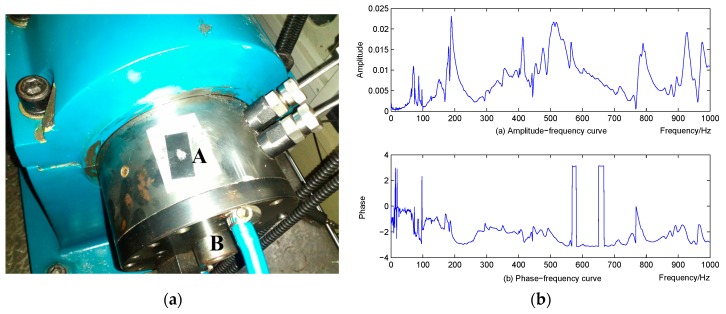
Spindle and tested FRF (**a**) spindle (**b**) FRF.

A spindle is taken as the control object in this simulation, as shown in Figure 6a. Piezoelectric patches (M-8557-P1, M + P, Hanover, Germany) are supposed fixed on point A and the acceleration sensor is fixed on point B. Frequency Response Function (FRF) is tested and used to simulate the controlled spindle (Figure 6b). The control flow runs in the following steps. First, the spindle is actuated by the simulated sinusoidal signal, and the responses are collected through FRF. Then the vibration is collected by the acceleration sensor and transferred to the frequency domain by FFT and fed back to the LMS control algorithm after comparing with the target signal. Next, the control algorithm will optimize a series of control parameters and send them as input to the FRF of the piezoelectric patch which can be found in the M + P handbook. Finally, the simulated spindle will be actuated by the original vibration source and the piezoelectric patch together, and the vibration responses will be judged by the hybrid error criterion. This process will be repeated until the hybrid error criterion is satisfied.

**Figure 7:**
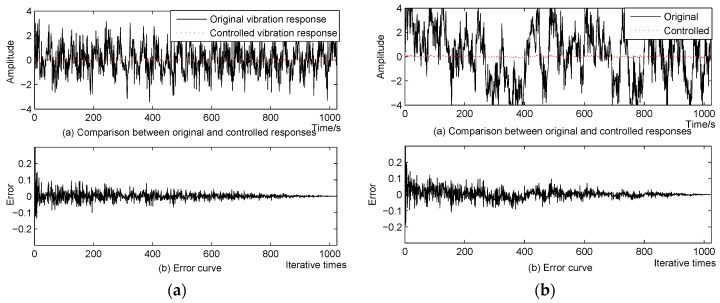
Control result of spindle actuated by: (**a**) single frequency sinusoidal signal (**b**) multifrequency sinusoidal signal.

The control algorithm is tested in this simulation in two cases. First, the simulated spindle is actuated by the original vibration source with a single frequency sinusoidal signal with white noise. The control results and the error curve can be seen in Figure 7a. Next, the original vibration source with a multifrequency sinusoidal signal is tested, and the control results can be seen in Figure 7b. It can be seen from these two figures that the proposed method reached well the control destination. However, if the original vibration source is a single frequency sinusoidal signal, the control process is more efficient and smooth, whereas if the vibration source has a multifrequency signal, the control process will be somewhat more complex, and will oscillate slightly. However, for either the single frequency or multifrequency case, the proposed active vibration suppression method performs very well and achieves satisfactory results.

### 3.2. Milling Machine Tool Based Experiment

**Figure 8:**
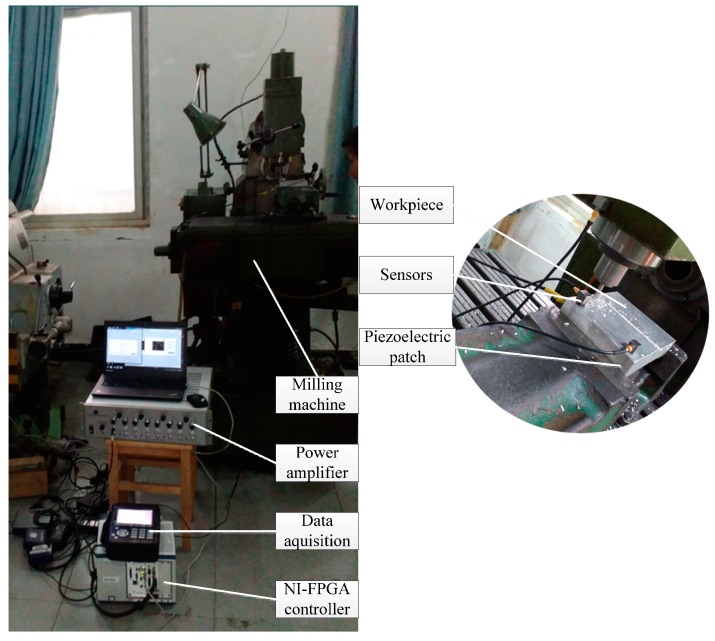
Set-up of the milling process experimental system.

**Figure 9:**
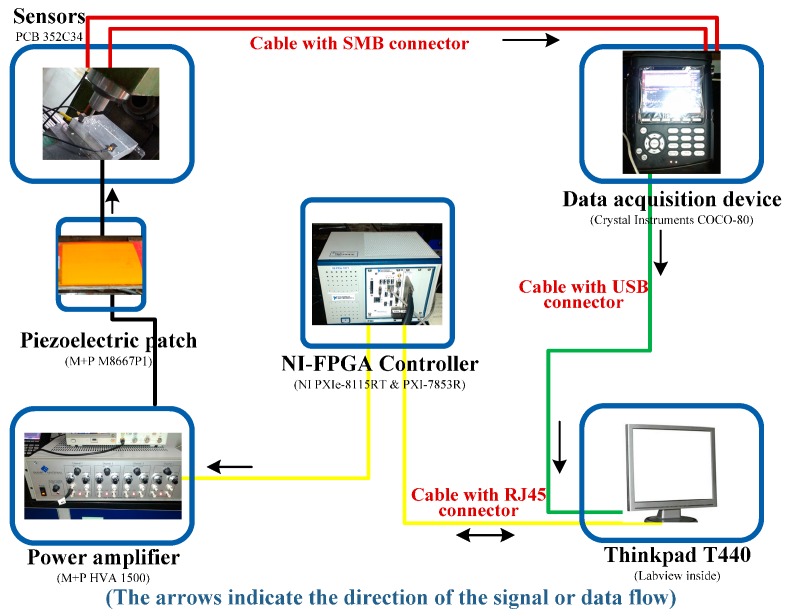
Data flow of the milling process experiment.

The experimental set-up of the milling process is composed of six parts, namely, the milling machine tool, workpiece, sensors, data acquisition device, NI-FPGA controller, power amplifier and piezoelectric patch, as shown in Figure 8. The sampling rate in this experiment is set as 10,240 Hz. Due to its excellent real time performance, the commercial real-time NI PXI-7853R FPGA controller [28] has been selected for on-line control and vibration suppression. The proposed frequency-domain LMS control algorithm in Figure 2 is programed by Labview and downloaded to the FPGA controller to optimize the actuating parameters. As the data flow shows in Figure 9, on-line vibration responses are first collected by sensors and the COCO-80 data acquisition device. Then the control parameters are optimized by the LMS control algorithm in FPGA and displayed on the computer. Next, the optimized actuating parameters are sent to the piezoelectric patch after being amplified by the power amplifier. This control process is repeated until the vibration responses in the milling process satisfy a preset target value.

The detailed information of this hardware is:

- Sensor (352C34, PCB, Depew, NY, USA)
  ➢ Sensitivity: (±10%) 100 mV/g (10.2)
  ➢ Measurement range: ±50 g pk (±490m/s^2^ pk)
  ➢ Broadband resolution: 0.00015 g rms (0.0015 m/s^2^ rms)
  ➢ Frequency range: (±5%) 0.5 to 10,000 Hz
- Piezoelectric patch (M-8557-P1, M + P)
  ➢ Active length 85 mm, active width 57 mm
  ➢ Capacitance 9.3 nF
  ➢ Free strain 1800 ppm
  ➢ Blocking force 923 N
- Control platform (PXIe-8115RT, NI)
  ➢ 2.5 GHz dual-core Intel Core i5-2510E processor
  ➢ 2GB (1 × 2 GB DIMM) single-channel 1333 MHz DDR3 RAM standard, 4 GB maximum
  ➢ 10/100/1000 BASE-TX (gigabit) Ethernet, ExpressCard/34 slot
  ➢ 191 kHz single PID loop rate, maximum
  ➢ Integrated hard drive, GPIB, serial, and other peripheral I/O
- NI FPGA controller (PXI-7853R, NI)
  ➢ User-defined triggering, timing, and decision making in hardware with 25 ns resolution
  ➢ Up to eight analog inputs, independent sampling rates up to 750 kHz, 16-bit resolution
  ➢ Up to eight analog output, independent update rate up to 1 MHz, 16-bit resolution
  ➢ Up to 160 digital lines configurable as inputs, output or counters at rate up to 40 MHz
  Direct memory access channels for data streaming
- Power amplifier (HVA1500, M + P)
  ➢ Up to four independent channels
  ➢ Voltage: up to 1500 V
  ➢ Designed for precise control of single MFC actuators and MFC actuator arrays
- Data acquisition device (COCO-80, CI, Santa Clara, CA, USA)
  ➢ Inputs: Two to eight BNC connectors with voltage or IEPE
  ➢ Outputs: 1 SMB connector, 100 dB dynamic range, 24-bit D/A converter
  ➢ Maximum sampling rate: 102.4 kHz simultaneously
  ➢ Flash memory: 4 GB used for system and data storage

**Figure 10:**
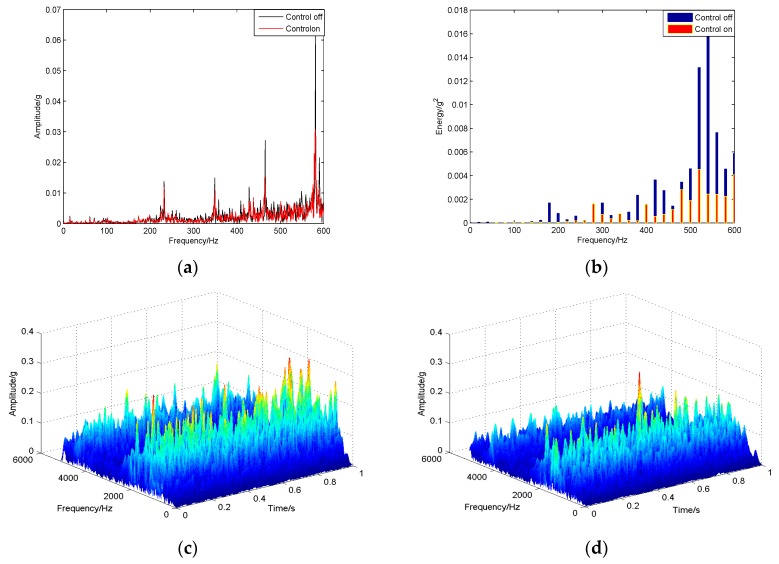
Experimental results of active vibration suppression in a milling process: (**a**) frequency spectrum (**b**) energy histogram (**c**) time-frequency waterfall diagram with control off (**d**) time-frequency waterfall diagram with control on.

**Figure 11:**
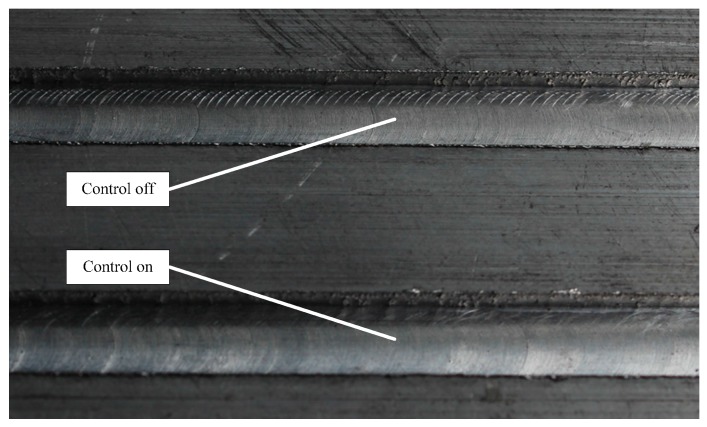
Machining effect of the aluminium plate with and without on-line vibration suppression.

In this experiment, the milling machine cuts two grooves on the aluminium plate with the following cutting parameters: speed 585 r/min; feed rate 60 mm/min and cut depth 2 mm. The experimental vibration responses are analyzed and compared in Figure 10 by frequency spectrum, energy histograms and time-frequency waterfall diagram. It can be seen from these plots or comparisons that the vibration amplitude or energy is reduced obviously when the constructed active control method is on, especially the low-frequency range energy histograms in which the the original value of the controlled vibration energy is reduced by more than 50%. The machining effect is shown in Figure 11. Aluminum plate is cut with two grooves, in which one is cut with the active control system on and one with the control system off. It can be seen that the second groove with the control system on is obviously better than the first one, whose surface is rougher. Therefore, the constructed active control algorithm is effective and useful in on-line manufacturing process, and it can improve surface smoothness in manufacturing.

## 4. Conclusions

Based on the classical LMS, a frequency-domain active control method is constructed for on-line vibration suppression of milling processes to improve the surface smoothness of the workpiece. A hybrid error criterion is constructed by combining the global frequency error and frequency node error together, thus the adaptability, anti-interference ability and efficiency of this method is improved. Spindle-based simulation and milling machine-based experiments are introduced to verify the proposed method and control platform. In the experiment, taking aluminium plate as control object, two grooves are cut by a milling machine, one with the control on and one in the natural condition. Analysis of the results, including frequency spectrum, energy histograms and time-frequency waterfall diagram, indicates that the proposed hybrid error criterion-based frequency-domain active vibration suppression method can achieve satisfactory effects in on-line milling processes. The machining effect of the aluminium plate also proves this point again.

Vibration is only one of the factors affects the machining quality of workpieces. Faults are another threat to machining quality, and may lead to unpredictable consequences. Therefore, in order to guarantee the reliability and machining effect, we plan to integrate together the condition monitoring, fault diagnosis and active vibration suppression units into machine tools to improve the performance of machine tools.
